# Supplementary material for: The effect of clinically elevated body mass index on physiological stress during manual lifting activities
Source: PLoS One. 2022 Dec 28;17(12):e0278858. doi: 10.1371/journal.pone.0278858 (PMC9797066; doi:10.1371/journal.pone.0278858)

PLOS ONE

THE EFFECT OF CLINICALLY ELEVATED BODY MASS INDEX ON PHYSIOLOGICAL STRESS DURING MANUAL LIFTING ACTIVITIES

--Manuscript Draft--

**Manuscript Number:**	PONE-D-22-28339
**Article Type:**	Research Article
**Full Title:**	THE EFFECT OF CLINICALLY ELEVATED BODY MASS INDEX ON PHYSIOLOGICAL STRESS DURING MANUAL LIFTING ACTIVITIES
**Short Title:**	PHYSIOLOGICAL STRESS DURING MANUAL LIFTING ACTIVITIES
**Corresponding Author:**	Francesco Travascio, Ph.D.<br>University of Miami - Coral Gables Campus: University of Miami<br>Coral Gables, Florida UNITED STATES
**Keywords:**	Obesity; manual material handling; revised NIOSH lifting equation (RNLE); energy expenditure; recommended weight limit (RWL)
**Abstract:**	Individuals with a body mass index (BMI) above normal constitute more than one-third of the Transportation, Warehousing, and Utilities (TWU) sector. These overweight and obese workers are more likely to experience work-related injuries. However, ergonomists still design work tasks based on the general population and normal body weight. This is particularly true for manual lifting tasks and the calculation of recommended weight limits (RWL) as per NIOSH guidelines. This study investigates the effects of BMI on indicators of physiological stress. It was hypothesized that, for clinically elevated BMI individuals, repeated manual lifting at RWL would produce physiological stress above safety limits. A repetitive box lifting task was designed to measure metabolic parameters: volume of carbon dioxide (VCO2) and oxygen (VO2), respiratory exchange ratio (RER), heart rate (HR), and energy expenditure rate (EER). A two-way ANOVA compared metabolic variables with BMI classification and gender, and linear regressions investigated BMI correlations. Results showed that BMI classification represented a significant effect for four parameters: VCO2 ($p < 0.001$), VO2 ($p < 0.001$), HR ($p = 0.012$), and EER ($p < 0.001$). In contrast, gender only had a significant effect on VO2 ($p = 0.014$) and EER ($p = 0.017$). Furthermore, significant positive relationships were found between BMI and VCO2 (R2 = 59.65%, $p < 0.001$), VO2 (R2 = 45.01%, $p < 0.001$), HR (R2 = 21.86%, $p = 0.009$), and EER (R2 = 50.83%, $p < 0.001$). Importantly, 80% of obese subjects exceeded the EER safety limit of 4.7 kcal/min indicated by NIOSH. Indicators of physiological stress are increased in clinically elevated BMI groups and appear capable of putting these workers at increased risk for workplace injury.
**Order of Authors:**	Sergio A Lemus, B.S.
	Mallory Volz, B.S.
	Eduard Tiozzo, Ph.D.
	Arlette Perry, Ph.D.
	Thomas M Best, M.D. Ph.D.
	Francesco Travascio, Ph.D.
**Additional Information:**	
**Question**	**Response**
**Financial Disclosure**<br><br>Enter a financial disclosure statement that describes the sources of funding for the work included in this submission. Review the submission guidelines for detailed requirements. View published research	FT and ET received funds from The University of Miami (award UM-PRA-2022-3243) to complete this study. The funders had no role in study design, data collection and analysis, decision to publish, or preparation of the manuscript.

Powered by Editorial Manager® and ProduXion Manager® from Aries Systems Corporation

articles from *PLOS ONE* for specific examples.<br><br>This statement is required for submission and **will appear in the published article** if the submission is accepted. Please make sure it is accurate.<br><br>**Unfunded studies**<br>Enter: *The author(s) received no specific funding for this work.*<br><br>**Funded studies**<br>Enter a statement with the following details:<br>• Initials of the authors who received each award<br>• Grant numbers awarded to each author<br>• The full name of each funder<br>• URL of each funder website<br>• Did the sponsors or funders play any role in the study design, data collection and analysis, decision to publish, or preparation of the manuscript?<br>• **NO** - Include this sentence at the end of your statement: *The funders had no role in study design, data collection and analysis, decision to publish, or preparation of the manuscript.*<br>• **YES** - Specify the role(s) played.<br><br>* typeset	
**Competing Interests**<br><br>Use the instructions below to enter a competing interest statement for this submission. On behalf of all authors, disclose any competing interests that could be perceived to bias this work—acknowledging all financial support and any other relevant financial or non-financial competing interests.<br><br>This statement is required for submission and **will appear in the published article** if the submission is accepted. Please make sure it is accurate and that any funding sources listed in your Funding Information later in the submission form are also declared in your Financial Disclosure statement.	The authors have declared that no competing interests exist.

View published research articles from *PLOS ONE* for specific examples.<br><br>**NO authors have competing interests**<br><br>Enter: *The authors have declared that no competing interests exist.*<br><br>**Authors with competing interests**<br><br>Enter competing interest details beginning with this statement:<br><br>*I have read the journal's policy and the authors of this manuscript have the following competing interests: [insert competing interests here]*<br><br>* typeset	
**Ethics Statement**<br><br>Enter an ethics statement for this submission. This statement is required if the study involved:<br><br>• Human participants<br>• Human specimens or tissue<br>• Vertebrate animals or cephalopods<br>• Vertebrate embryos or tissues<br>• Field research<br><br>Write "N/A" if the submission does not require an ethics statement.<br><br>General guidance is provided below. Consult the submission guidelines for detailed instructions. **Make sure that all information entered here is included in the Methods section of the manuscript.**	The Institutional Review Board of the University of Miami authorized this study (IRB ID: 20211175). Written consent was obtained from participants.

Powered by Editorial Manager® and ProduXion Manager® from Aries Systems Corporation

**Format for specific study types**<br><br>**Human Subject Research (involving human participants and/or tissue)**<br>• Give the name of the institutional review board or ethics committee that approved the study<br>• Include the approval number and/or a statement indicating approval of this research<br>• Indicate the form of consent obtained (written/oral) or the reason that consent was not obtained (e.g. the data were analyzed anonymously)<br><br>**Animal Research (involving vertebrate animals, embryos or tissues)**<br>• Provide the name of the Institutional Animal Care and Use Committee (IACUC) or other relevant ethics board that reviewed the study protocol, and indicate whether they approved this research or granted a formal waiver of ethical approval<br>• Include an approval number if one was obtained<br>• If the study involved *non-human primates*, add *additional details* about animal welfare and steps taken to ameliorate suffering<br>• If anesthesia, euthanasia, or any kind of animal sacrifice is part of the study, include briefly which substances and/or methods were applied<br><br>**Field Research**<br><br>Include the following details if this study involves the collection of plant, animal, or other materials from a natural setting:<br>• Field permit number<br>• Name of the institution or relevant body that granted permission	
**Data Availability**<br><br>Authors are required to make all data underlying the findings described fully available, without restriction, and from the time of publication. PLOS allows rare exceptions to address legal and ethical concerns. See the PLOS Data Policy and FAQ for detailed information.	Yes - all data are fully available without restriction

A Data Availability Statement describing where the data can be found is required at submission. Your answers to this question constitute the Data Availability Statement and **will be published in the article**, if accepted.<br><br>**Important:** Stating 'data available on request from the author' is not sufficient. If your data are only available upon request, select 'No' for the first question and explain your exceptional situation in the text box.<br><br>Do the authors confirm that all data underlying the findings described in their manuscript are fully available without restriction?	
**Describe where the data may be found in full sentences. If you are copying our sample text, replace any instances of XXX with the appropriate details.**<br><br>• If the data are **held or will be held in a public repository**, include URLs, accession numbers or DOIs. If this information will only be available after acceptance, indicate this by ticking the box below. For example: *All XXX files are available from the XXX database (accession number(s) XXX, XXX.).*<br>• If the data are all contained **within the manuscript and/or Supporting Information files**, enter the following: *All relevant data are within the manuscript and its Supporting Information files.*<br>• If neither of these applies but you are able to provide **details of access elsewhere**, with or without limitations, please do so. For example:<br><br>*Data cannot be shared publicly because of [XXX]. Data are available from the XXX Institutional Data Access / Ethics Committee (contact via XXX) for researchers who meet the criteria for access to confidential data.*<br><br>*The data underlying the results presented in the study are available from (include the name of the third party*	All relevant data are within the manuscript and its Supporting Information files

and contact information or URL).<br>• This text is appropriate if the data are owned by a third party and authors do not have permission to share the data.<br><br>* typeset	
Additional data availability information:	

Manuscript
Click here to access/download;Manuscript;main manuscript.docx

THE EFFECT OF CLINICALLY ELEVATED BODY MASS INDEX ON PHYSIOLOGICAL STRESS DURING MANUAL LIFTING ACTIVITIES

Sergio A. Lemus[1], Mallory Volz[2], Eduard Tiozzo[3], Arlette Perry[4,5,6], Thomas M. Best[2,7,8], Francesco Travascio[1,8,9*]

[1]Department of Mechanical and Aerospace Engineering, University of Miami, Coral Gables, FL
[2]Department of Biomedical Engineering, University of Miami, Coral Gables, FL
[3]Department of Physical Medicine and Rehabilitation, University of Miami, Miami, FL
[4]Department of Kinesiology and Sport Sciences, University of Miami, Coral Gables, FL
[5]Laboratory of Clinical and Applied Physiology, University of Miami, Coral Gables, FL
[6]School of Education and Human Development, University of Miami, Coral Gables, FL
[7]Department of Orthopaedic Surgery, University of Miami, Miami, FL
[8]UHealth Sports Medicine Institute, Coral Gables, FL
[9]Max Biedermann Institute for Biomechanics at Mount Sinai Medical Center, Miami Beach, FL

Submitted to PLoS One October 13th, 2022

Running Title: PHYSIOLOGICAL STRESS DURING MANUAL LIFTING ACTIVITIES

***Corresponding Author:**
Francesco Travascio, PhD
Associate Professor
College of Engineering
University of Miami
1251 Memorial Drive, MEB 276
Coral Gables, FL, 33146
USA
Telephone: +1-(305)-2842371
Email : f.travascio@miami.edu

Eduard Tiozzo, Ph.D. MSCTI FAHA
Research Assistant Professor
Miller School of Medicine
University of Miami
NE 5th Street
Miami, FL 33132
USA
Telephone: +1-(305)-2431633
Email : e.tiozzo@med.miami.edu

Acknowledgments
The project described was supported by Grant Number UM-PRA-2022-3243 from the University of Miami.

ABSTRACT

Individuals with a body mass index (BMI) above normal constitute more than one-third of the Transportation, Warehousing, and Utilities (TWU) sector. These overweight and obese workers are more likely to experience work-related injuries. However, ergonomists still design work tasks based on the general population and normal body weight. This is particularly true for manual lifting tasks and the calculation of recommended weight limits (RWL) as per NIOSH guidelines. This study investigates the effects of BMI on indicators of physiological stress. It was hypothesized that, for clinically elevated BMI individuals, repeated manual lifting at RWL would produce physiological stress above safety limits. A repetitive box lifting task was designed to measure metabolic parameters: volume of carbon dioxide (VCO_2) and oxygen (VO_2), respiratory exchange ratio (RER), heart rate (HR), and energy expenditure rate (EER). A two-way ANOVA compared metabolic variables with BMI classification and gender, and linear regressions investigated BMI correlations. Results showed that BMI classification represented a significant effect for four parameters: VCO_2 ($p < 0.001$), VO_2 ($p < 0.001$), HR ($p = 0.012$), and EER ($p < 0.001$). In contrast, gender only had a significant effect on VO_2 ($p = 0.014$) and EER ($p = 0.017$). Furthermore, significant positive relationships were found between BMI and VCO_2 ($R^2 = 59.65\%$, $p < 0.001$), VO_2 ($R^2 = 45.01\%$, $p < 0.001$), HR ($R^2 = 21.86\%$, $p = 0.009$), and EER ($R^2 = 50.83\%$, $p < 0.001$). Importantly, 80% of obese subjects exceeded the EER safety limit of 4.7 kcal/min indicated by NIOSH. Indicators of physiological stress are increased in clinically elevated BMI groups and appear capable of putting these workers at increased risk for workplace injury.

2. INTRODUCTION

In most occupational and industry groups, the prevalence of injury is positively correlated to the proportion of high body mass index (BMI) individuals comprising the workforce [1-3]. This is especially true for the Transport Warehousing and Utilities (TWU) Super Sector which, among all occupational groups, reports the highest number of musculoskeletal injuries [4]. Approximately 1/3 of the TWU Super Sector workforce is characterized by a BMI larger than average [5]. These overweight and obese workers are 26% to 45% more likely to experience work related injuries, when compared to average BMI individuals [6]. Numerous causal and mechanistic relationships between the clinically elevated BMI population and increased risk of injury have been proposed [7]. For example, obesity is associated with a decrease in capillary density and reduced blood flow to skeletal muscle [8], limiting the supply of oxygen and nutrients to working tissue. This can potentially result in a faster onset of muscle fatigue during sustained exertions [9]. In addition, the muscular fiber composition of obese people has been shown to have a lower percentage of fatigue resistant fibers when compared to average BMI individuals [10]. It is also already established that obese people have a higher cardiovascular demand when performing basic occupational tasks, due to reduced metabolic and conditioning performance [11-14].

Despite the adverse effects of clinically elevated BMI on the prevalence of musculoskeletal injuries in blue collar workers, workspace and work tasks are still being designed using anthropometric characteristics derived from average “healthy” workers [15, 16]. Such is the case for manual lifting tasks that account for a major portion of activities in the TWU sector [17]. Sound ergonomic practice suggests that such tasks should be designed based on the revised National Institute for Occupational Safety and Health (NIOSH) lifting equation (RNLE) [18]. Specifically, the RNLE is an ergonomic tool designed to reduce the physical stress in manual lifting activities

[18]. Developed on the basis of both physiological and biomechanical safety criteria, the RNLE calculates recommended weight limits (RWL) with the purpose of safely regulating the energy expenditure and musculoskeletal loads during manual lifting [19]. The RNLE comprises experimental coefficients (multipliers) accounting for work geometry (e.g., initial. and final location of the object to be lifted, etc.), frequency, and duration of the task. According to the RNLE, the RWL is modulated according to the frequency and duration of the lifting task in order maintain a person's energy expenditure within a safety limit of 4.7 kcal/min [19]. Furthermore, the RNLE multipliers are based on experimental data obtained from an average BMI population [15, 16]. In other words, the calculation of RWL does not explicitly account for effects of BMI. Consequently, we hypothesized that, for large BMI individuals, the execution of manual lifting at RWL would produce a level of physiological stress above safety limits. Therefore, the aim of this study was to measure and compare metabolic parameters indicative of physiological stress in normal weight, overweight, and obese individuals while performing manual lifting activities at RWL.

2. METHODS

A repetitive box lifting task was designed to measure metabolic outputs and explore the effects of BMI on physiological stress. Subjects lifted a box from the floor to the table and back at a predetermined frequency while metabolic measurements were conducted. Subject recruitment, work geometry, and detailed experimental procedures are described below.

2.1 Subjects recruitment

The methods used in this cross-sectional study were approved by the Internal Review Board of the University of Miami. All participants were informed of the study procedures, provided written consent prior to beginning the experiment, and were given the option to withdraw at any time. Participants were recruited from the Miami-Dade metropolitan area through advertisement flyers posted in common areas on and surrounding the University's main campus, as well as Facebook posts in local groups. The enrollment consisted of 30 people stratified into groups of 10 (5 males and 5 females with gender being self-reported) for 3 BMI classifications based on the definitions of the World Health Organization [20]: normal weight (18.5 ≤ BMI ≤ 24.9), overweight (25 ≤ BMI ≤ 29.9), and obese (BMI ≥ 30). A single subject with a BMI of 16.71 was determined to not be statistically different from the metabolic measures of the normal classification and thus was grouped as such. Ethnic and racial composition of subjects was chosen to represent, as close as possible, Miami-Dade County demographics [21]. Exclusion criteria for the study consist of pregnancy, history of musculoskeletal injuries in the past 6 months or any chronic cardiopulmonary-related conditions that may be exacerbated during tests (including but not limited to asthma, chronic obstructive pulmonary disease, congestive heart failure, and/or lung disease).

2.2 Work geometry and lifting task

Subjects stood in front of a box (10”x10”x10”) and moved it from the floor to the 30” high table, and from the table to the floor. The box was lifted at a frequency of six lifts per minute for 20 minutes, which is considered a moderate exercise intensity based on the threshold guidelines for five intensity zones (very light, light, moderate, vigorous, near maximal/maximal) [22]. The hand grasps of the box were 10” away from the midpoint of the subject’s ankles and 5” from the floor. A ‘freestyle’ lifting technique was allowed. The geometrical setting of the task is shown in Figure 1. The weight of the box was chosen to be equivalent to the RWL as defined in the revised NIOSH lifting equation RNLE:

$$RWL = LC \times HM \times VM \times DM \times AM \times FM \times CM \text{ [18],}$$

Where LC is a load constant equal to 51 pounds; the multipliers HM, VM and DM account for the horizontal and vertical location of the load with respect to the operator, and the vertical distance the load during lifting; the multiplier AM accounts for trunk rotation while lifting (asymmetry); FM varies according to frequency and duration of the lifting task; CM accounts for the hand-to-object coupling [18]. The specific numerical values of the RNLE multipliers, depending on the specific work geometry and the frequency of lifting, are reported in Table 2. The resulting RWL for the specific task investigated was 27.5 ~~pounds.~~

2.3 Metabolic measurements

Metabolic data during the lifting task were acquired via a CardioCoachCO_2 metabolic cart (KORR, Utah, USA) in conjunction with a heart rate monitor (Polar, Kempele, Finland). After a standardized automatic calibration, the equipment measured the volume of carbon dioxide production (VCO_2), volume of oxygen consumption (VO_2), and heart rate (HR). The values of VO_2 and VCO_2 were used to compute the respiratory exchange ratio (RER), which refers to the ratio of

CO_2 production to O_2 consumption and indirectly shows skeletal muscle's oxidative capacity to obtain energy [23]:

$$RER = \frac{VCO_2}{VO_2} \ (1).$$

In addition, the rates of oxygen consumption (VO_2) and carbon dioxide production (VCO_2) were used to determine the energy expenditure rate (EER) according to the Weir equation [24]:

$$EER\left[\frac{kcal}{min}\right] = 3.94 \cdot VO_2\left[\frac{ml}{min}\right] + 1.11 \cdot VCO_2\left[\frac{ml}{min}\right] \ (2)$$

Where the coefficients 3.94 and 1.11 relate the volume of gas exchange to the calories consumed per minute.

2.4 Statistical analysis

Time-dependent measurements for VCO_2, VO_2, RER, HR, and EER for each BMI group were reported with sampling at 15 second intervals (Excel 2207, Microsoft Corporation, Washington, USA, and MATLAB (R2022a, MathWorks Inc., Massachusetts, USA). A five-point moving average was used to smooth the data points. When investigating statistical differences across metabolic parameters, the values of VCO_2, VO_2, RER, HR, and EER were those attained at steady state, according to the recommendations of indirect calorimetry published by The Academy of Nutrition and Dietetics (AND): after a 5-minute stabilization period, the steady state was determined when the coefficient of variation was less than 10% for all the metabolic parameters [25]. It has been reported that, at moderate exercise, steady state can be reached after 10-15 minutes [26-28]. In this study, the timeframe of observation was 20 minutes to guarantee full attainment of steady-state conditions.

For all the subjects groups investigated, the metabolic parameters went through a transient period, varying from 5 to 10 minutes, in which their magnitudes changed (increasing for VCO_2, VO_2, HR and EER, and decreasing for RER); the final 5-10 minutes of the observational period were characterized by a steady state. The subsequent statistical analysis was conducted in Minitab® (21.1.1, Minitab LLC, Pennsylvania, USA). A two-way ANOVA followed by Fisher Pairwise comparison was performed to compare the steady-state values of the metabolic variables, with factors being the BMI classification (three levels) and gender (two levels). Additionally, simple linear regression models investigated the correlation of BMI with steady-state values of VCO_2, VO_2, RER, HR, and EER. Grubb's tests were performed to detect outliers. For all the statistical analyses performed, significance was set at 95% ($\alpha = 0.05$). Finally, a post-hoc power analysis was conducted using G*Power version 3.1.9.7 [29] to find the statistical power for muscle activation considering muscle group as the main effect.

3. RESULTS

The sample (n = 30) comprised of 50% males (n = 15) and 50% females (n = 15) with a mean age of 32.5 (SD = 12.3) and BMI of 28.5 (SD = 7.43). The overall racial/ethnic distributions of the subjects were as follows: 40.0% Hispanic/Latino (n = 12), 16.6% white, not Hispanic/Latino (n = 5), 10% black, or African American (n = 3), 16.6% Asian (n = 5), and 16.6% two or more races/ethnicities (n = 5). The overall workforce distribution of the subjects was as follows: 53% education (n = 16), 20% service (n = 6), 20% management and sales (n=6), and 7% healthcare (n=2). A further breakdown of this anthropometric and demographic data across both BMI classifications and gender is available in Table 1.

The values of the metabolic parameters (VCO_2, VO_2, RER, HR, and EER) across the entire observational period of 20 minutes are reported in Figure 2. Data show that the magnitudes of RER were similar across all BMI groups, see Figure 2C. In contrast, the general trend of the other metabolic parameters indicated that obese and overweight subjects experienced higher values of VCO_2, VO_2, HR and EER, when compared to the normal BMI participants. Importantly, after the first 5 minutes of lifting, the values of EER of normal (3.8 kcal/min) and overweight (4.3 kcal/min) subjects remained below the NIOSH safety limit (4.7 kcal/min), while that of the obese reached 5.6 kcal/min, surpassing the safety threshold by 0.9 kcal/min (19%).

Statistical comparisons of magnitudes of metabolic parameters at steady state across gender and BMI groups are reported in Table 3. First, for each parameter investigated, no significant interaction between BMI classification and gender was found. However, gender produced a significant main effect for VO_2 ($p = 0.014$) and EER ($p = 0.017$): the magnitudes of these parameters found in men were higher than those of women. Post-hoc analysis showed that the power in determining statistical differences between genders for both VO_2 and EER was larger than 95%. Also, BMI classification was a significant

main effect for the four metabolic parameters VCO_2 ($p < 0.001$), VO_2 ($p < 0.001$), HR ($p = 0.012$) and EER ($p < 0.001$): a Fisher Pairwise comparison indicated that the magnitude of afore mentioned parameters for obese subjects was larger than those observed for other BMI groups. Post-hoc power analysis indicated power equal or larger than 95% when inferring statistical differences in VO_2, VCO_2, EER, and HR across BMI groups.

Moreover, simple linear regression models relating BMI to metabolic parameters were explored, and the results are reported in Figure 3. Significant positive relationships between subjects' BMI and VCO_2 ($R^2 = 59.65\%$, $p < 0.001$), VO_2 ($R^2 = 45.01\%$, $p < 0.001$), and HR ($R^2 = 21.86\%$, $p = 0.009$) were found. This was not the case for RER ($R^2 = 1.53\%$, $p = 0.515$). Importantly, BMI had a significant impact on EER ($R^2 = 50.83\%$, $p < 0.001$), with 80% of obese subjects exceeding the NIOSH safety limits, see Figure 3E.

4. DISCUSSION

Previous studies have identified a need to consider physiological implications in calculating RWL for all populations [30]. Barim et al. showed that the inclusion of new physiological RNLE multipliers, one of which was BMI, improved risk assessment [31]. Still, the effect of BMI on metabolic parameters during repeated manual lifting tasks has not been quantified. To the authors' best knowledge, this is the first study measuring and comparing the effects of clinically elevated BMI on indicators of physiological stresses at RWL. The overall results of this study indicate that obese subjects exceeded the NIOSH safety limit for EER during this lifting task.

Steady state values of VCO_2 and VO_2 provide crucial information on respiratory gas exchange during exercise. In the present study, the magnitudes of these parameters were significantly dependent on BMI group ($p < 0.001$): VCO_2 values associated with obese subjects were 27.0% higher than overweight subjects and 38.1% higher than normal subjects; VO_2 values associated with obese subjects were 25.8% higher than overweight subjects and 40.0% higher than normal subjects. The higher values of VCO_2 and VO_2 for obese people in the present study may be due to several reasons. First, obese people have a higher cardiac stroke volume and a higher mechanical demand on the lungs due to increased weight bearing [32-34]. They also suffer from metabolic inefficiencies and in many cases a low grade systemic inflammation that can create a higher request for oxygen during exercise [35, 36], resulting in increased respiratory rates for a given workload [37]. Finally, previous studies report that resting VCO_2 and VO_2 are naturally higher in people of clinically elevated BMI classified as obese [36, 38]. These gas volumes are shown to proportionally increase progressively from resting levels to intense exercise [36-38]. The increased inspiratory and expiratory gas volumes observed in obese subjects compensate for reduced cardiopulmonary

efficiency and are associated with increased adipose tissue, and these effects are only exacerbated by exercise as the work of breathing is increased.

RER is another metabolic parameter that is used to relate VCO_2 and VO_2 when lifting at RWL. In the present study, all genders and BMI classifications performed similarly, achieving a steady state RER between 0.82 and 0.84. Thus, the increases in VCO_2 and VO_2 associated with higher BMI groups are uniform and result in similar RER values across BMI groups. This finding is in agreement with previous studies showing no relationship between exercise RER and absent body fat [39]. Furthermore, typical values for exercise RER vary between 0.7 and 1.0, which gives insight into the energy source used to replenish ATP [36, 38]. Values close to 0.7 reflect lipid oxidation (mainly during lower intensity exercise), while values closer to 1.0 reflect carbohydrate oxidation (mainly during higher intensity exercise) [36, 38]. Thus, all subjects in the present study relied upon both lipid and carbohydrate oxidation to perform lifting tasks. However, lipid oxidation was favored; this is a reasonable finding for a moderate intensity activity [36, 38].

In contrast to RER, the higher values of VCO_2 and VO_2 in the obese and overweight BMI groups resulted in a significant effect on EER. The obese group had a 26.3% higher EER than the overweight group and 38.3% higher EER than the normal group. This is likely because VCO_2 and VO_2 are linear factors in Weir's equation, see Equation 2 [40]. Previous studies reported that obese people have both a greater basal metabolic rate and physical activity cost than lower BMI people [41, 42]. DeLany et al. suggested that these differences were due to the obese population generally having biomechanical inefficiencies that cause them to work harder and thus have a higher metabolic cost [41, 43]. These metabolic inefficiencies, in turn, contribute to a higher oxygen demand for a given workload seen in clinically elevated high BMI individuals during exercise [35, 36]. Similar to the components that contribute to VCO_2 and VO_2, exercise EER is likely increased

for the higher BMI groups because of the larger oxygen demand and inherent inefficiencies associated with increased body weight.

Furthermore, Figure 2C depicts the differences in EER between BMI groups in reference to the NIOSH safety limit. The EER of the obese group (5.6 kcal/min) surpassed the safety threshold (4.7 kcal/min), but this was not the case for overweight (4.3 kcal/min) and normal (3.8 kcal/min) groups. Specifically, Figure 3E shows that 80% of the tested obese subjects exceeded the NIOSH safety limit for EER during this lifting task. Overall, the relationship between EER and BMI confirms our hypothesis: for clinically elevated BMI individuals, manual lifting at RWL produced a level of physiological stress above safety limits established by the RNLE.

Similar to the EER, BMI class also significantly impacted HR, see Table 3. Obese subjects experienced higher HRs than both overweight and normal subjects. Notably, the obese subjects had a 20% higher HR than the other two BMI groups. Previous studies reported that obese people have an elevated resting HR [44-47], which puts them at increased risk for cardiometabolic problems [48-50]. Cardiorespiratory fitness, which is more common among people of lower BMI, has been associated with a decrease in resting heart rate [51, 52]. Moreover, heart rate increases from resting to exercise, and is linearly dependent on exercise intensity [53, 54]. These established findings explain the increased HR observed in subjects of higher BMI during the exercise activity in the present study.

To further explore the relationships between BMI and metabolism, simple linear regressions were explored, see Figure 3. There was a positive correlation between a subjects' BMI and VCO_2 (R^2 = 45.01%; $p < 0.001$), VO_2 (R^2 = 59.65%; $p < 0.001$), EER (R^2 = 50.83%; $p < 0.001$), and HR (R^2 = 21.86%; $p = 0.009$). However, BMI did not significantly influence RER (R^2

= 1.53%; p = 0.515). These results corroborate our findings on the effect of BMI on metabolic parameters (Table 3).

Gender also had some effect on certain metabolic variables. Males had 15.3% higher VO_2 (p = 0.014) and 15.4% higher EER (p = 0.017) than females. This relationship is in agreement with previous studies, which showed that men had a significantly greater VO_2 [55, 56] and EER [56, 57] than women during steady-state submaximal exercise. Thus, there are likely underlying physiological differences between males and females that are evidenced through VO_2 and EER values.

Some limitations should be acknowledged. A convenience sample of 30 subjects (15 male and 15 female) was used in the present study. While a larger pool of participants would be desirable, results from the post-hoc power analysis show that this sample size was large enough to observe effects of BMI and gender with power equal or larger than 95%. Also, our subjects had occupations that did not necessarily involve manual material handling, see Table 1C. Future studies should be conducted to investigate the effects of physiological stress on subjects specifically belonging to the TWU workforce. In addition, BMI does not take into account body fat percentage or distribution. While the researchers only selected obese participants with little to no reported exercise history, body fat could still be an influential variable that should be analyzed in the future. Furthermore, the repetitive lifting activity performed in this analysis only represents a single component of the many TWU tasks, and future effort should be devoted to investigating additional tasks that comprehensively reflect the TWU workplace environment. Still, the activity we used was conducted according to the NIOSH guidelines to enforce physiological safety limits. Thus, the present study can be used as a basis for understanding how clinically elevated BMI may impact a worker's risk for injury.

In summary, this study examined the effects of BMI on physiological stress during a manual lifting task designed according to the established safety guidelines of the RNLE. It was found that clinically elevated BMI led to an increase in VCO_2, VO_2, EER, and HR. Of note, obese subjects exceeded the NIOSH energy expenditure safety limit, and suggests that these individuals would be at increased risk for workplace injury as compared to their overweight and normal counterparts. The results of this study can be used to determine additional BMI multipliers for the RNLE, , to help reduce the risk of physiological stress, potentially leading to injury, for workers with a larger BMI. Future research should focus on larger populations for the purpose of establishing cut-points for increased BMI.

REFERENCES

1. Ostbye, T., J.M. Dement, and K.M. Krause, *Obesity and workers' compensation: results from the Duke Health and Safety Surveillance System.* Arch Intern Med, 2007. **167**(8): p. 766-73.
2. Schmier, J.K., M.L. Jones, and M.T. Halpern, *Cost of obesity in the workplace.* Scand J Work Environ Health, 2006. **32**(1): p. 5-11.
3. Janssen, I., E. Bacon, and W. Pickett, *Obesity and its relationship with occupational injury in the canadian workforce.* J Obes, 2011. **2011**: p. 531403.
4. Dunning, K.K., K.G. Davis, C. Cook, S.E. Kotowski, C. Hamrick, G. Jewell, et al., *Costs by industry and diagnosis among musculoskeletal claims in a state workers compensation system: 1999-2004.* Am J Ind Med, 2010. **53**(3): p. 276-84.
5. Helmkamp, J.C., J.E. Lincoln, J. Sestito, E. Wood, J. Birdsey, and M. Kiefer, *Risk factors, health behaviors, and injury among adults employed in the transportation, warehousing, and utilities super sector.* Am J Ind Med, 2013. **56**(5): p. 556-68.
6. Gu, J.K., L.E. Charles, D. Fekedulegn, C.C. Ma, M.E. Andrew, and C.M. Burchfiel, *Prevalence of Injury in Occupation and Industry: Role of Obesity in the National Health Interview Survey 2004 to 2013.* J Occup Environ Med, 2016. **58**(4): p. 335-43.
7. Cavuoto, L. and M. Nussbaum, *Influences of Obesity on Job Demands and Worker Capacity.* Current Obesity Reports, 2014. **3**.
8. Kern, P.A., R.B. Simsolo, and M. Fournier, *Effect of weight loss on muscle fiber type, fiber size, capillarity, and succinate dehydrogenase activity in humans.* J Clin Endocrinol Metab, 1999. **84**(11): p. 4185-90.
9. Newcomer, B.R., D.E. Larson-Meyer, G.R. Hunter, and R.L. Weinsier, *Skeletal muscle metabolism in overweight and post-overweight women: an isometric exercise study using*

(31)P magnetic resonance spectroscopy. Int J Obes Relat Metab Disord, 2001. **25**(9): p. 1309-15.

10. Maltin, C.A., *Muscle development and obesity: Is there a relationship?* Organogenesis, 2008. **4**(3): p. 158-69.
11. Cavuoto, L.A. and M.A. Nussbaum, *The influences of obesity and age on functional performance during intermittent upper extremity tasks.* J Occup Environ Hyg, 2014. **11**(9): p. 583-90.
12. Mehta, R.K. and L.A. Cavuoto, *The effects of obesity, age, and relative workload levels on handgrip endurance.* Appl Ergon, 2015. **46 Pt A**: p. 91-5.
13. Karas, B.E. and K.M. Conrad, *Back Injury Prevention Interventions in the Workplace: An Integrative Review.* AAOHN Journal, 1996. **44**(4): p. 189-196.
14. Xu, X., G.A. Mirka, and S.M. Hsiang, *The effects of obesity on lifting performance.* Appl Ergon, 2008. **39**(1): p. 93-8.
15. Capodaglio, P., G. Castelnuovo, A. Brunani, L. Vismara, V. Villa, and E.M. Capodaglio, *Functional limitations and occupational issues in obesity: a review.* Int J Occup Saf Ergon, 2010. **16**(4): p. 507-23.
16. Gordon, C.C. and B. Bradtmiller, *Anthropometric change: implications for office ergonomics.* Work, 2012. **41 Suppl 1**: p. 4606-11.
17. Bureau of Labor Statistics, U.S.D.o.L., Occupational Outlook Handbook, Hand Laborers and Material Movers,, 2022.
18. Waters, T.R., V. Putz-Anderson, and A. Garg, *Applications manual for the revised NIOSH lifting equation.* U.S. Department of Health and Human Services 1994. **94**(110).

19. Waters, T.R., V. Putz-Anderson, A. Garg, and L.J. Fine, *Revised NIOSH equation for the design and evaluation of manual lifting tasks.* Ergonomics, 1993. **36**(7): p. 749-76.

20. *Obesity: preventing and managing the global epidemic. Report of a WHO consultation.* World Health Organ Tech Rep Ser, 2000. **894**: p. i-xii, 1-253.

21. *Quick Facts: Miami-Dade County* United States Census Bureau 2020; Available from: https://www.census.gov/quickfacts/fact/table/miamidadecountyflorida/POP060210.

22. Riebe D, E.J., Liguori G, Magal M, *ACSM's guidelines for exercise testing and prescription. Tenth edit. (Wolters Kluwer: Philadelphia, PA).* 2018.

23. Ramos-Jiménez, A., R.P. Hernández-Torres, P.V. Torres-Durán, J. Romero-Gonzalez, D. Mascher, C. Posadas-Romero, et al., *The Respiratory Exchange Ratio is Associated with Fitness Indicators Both in Trained and Untrained Men: A Possible Application for People with Reduced Exercise Tolerance.* Clinical medicine. Circulatory, respiratory and pulmonary medicine, 2008. **2**: p. CCRPM.S449.

24. Weir, J.B.d.V., *New methods for calculating metabolic rate with special reference to protein metabolism.* The Journal of Physiology, 1949. **109**(1-2): p. 1-9.

25. Fullmer, S., S. Benson-Davies, C.P. Earthman, D.C. Frankenfield, E. Gradwell, P.S.P. Lee, et al., *Evidence Analysis Library Review of Best Practices for Performing Indirect Calorimetry in Healthy and Non–Critically Ill Individuals.* Journal of the Academy of Nutrition and Dietetics, 2015. **115**(9): p. 1417-1446.e2.

26. Jones, A.M. and H. Carter, *The Effect of Endurance Training on Parameters of Aerobic Fitness.* Sports Medicine, 2000. **29**(6): p. 373-386.

27. Åstrand, P.-O., T.E. Cuddy, B. Saltin, and J. Stenberg, *Cardiac output during submaximal and maximal work.* Journal of Applied Physiology, 1964. **19**(2): p. 268-274.

28. Jones, A.M., M. Burnley, M.I. Black, D.C. Poole, and A. Vanhatalo, *The maximal metabolic steady state: redefining the 'gold standard'*. Physiol Rep, 2019. **7**(10): p. e14098.

29. Faul, F., E. Erdfelder, A.G. Lang, and A. Buchner, *G*Power 3: a flexible statistical power analysis program for the social, behavioral, and biomedical sciences.* Behav Res Methods, 2007. **39**(2): p. 175-91.

30. Ahmad, S. and M. Muzammil. *Study of the Effect of Worker Characteristics on Maximum Acceptable Weight of Lift*. 2022. Cham: Springer International Publishing.

31. Barim, M.S., R.F. Sesek, M.F. Capanoglu, P. Drinkaus, M.C. Schall, S. Gallagher, et al., *Improving the risk assessment capability of the revised NIOSH lifting equation by incorporating personal characteristics.* Applied Ergonomics, 2019. **74**: p. 67-73.

32. Serés, L., J. López-Ayerbe, R. Coll, O. Rodríguez, J.M. Manresa, J. Marrugat, et al., *[Cardiopulmonary function and exercise capacity in patients with morbid obesity]*. Rev Esp Cardiol, 2003. **56**(6): p. 594-600.

33. Gonze, B.B., T. Ostolin, A.C.B. Barbosa, A.C. Matheus, E.F. Sperandio, A.R.T. Gagliardi, et al., *Dynamic physiological responses in obese and non-obese adults submitted to cardiopulmonary exercise test.* PLoS One, 2021. **16**(8): p. e0255724.

34. Brazzale, D.J., J.J. Pretto, and L.M. Schachter, *Optimizing respiratory function assessments to elucidate the impact of obesity on respiratory health.* Respirology, 2015. **20**(5): p. 715-721.

35. Santoro, C., R. Sorrentino, R. Esposito, M. Lembo, V. Capone, F. Rozza, et al., *Cardiopulmonary exercise testing and echocardiographic exam: an useful interaction.* Cardiovascular Ultrasound, 2019. **17**(1): p. 29.

36. *BSL Pro lesson H19: VO2, VCO2, RER, O2 deficit and EPOC measurement*, I. BIOPAC Systems, Editor. 2015.

37. Littleton, S.W., *Impact of obesity on respiratory function.* Respirology, 2012. **17**(1): p. 43-49.

38. Luks, A.M., R. Glenny, and H.T. Robertson, *Introduction to cadiopulmonary exercise testing*. Vol. 2. 2013: Springer.

39. Helge, J.W., A.M. Fraser, A.D. Kriketos, A.B. Jenkins, G.D. Calvert, K.J. Ayre, et al., *Interrelationships between muscle fibre type, substrate oxidation and body fat.* Int J Obes Relat Metab Disord, 1999. **23**(9): p. 986-91.

40. Matarese, L.E., *Indirect Calorimetry: Technical Aspects.* Journal of the American Dietetic Association, 1997. **97**(10): p. S154-S160.

41. DeLany, J.P., D.E. Kelley, K.C. Hames, J.M. Jakicic, and B.H. Goodpaster, *High energy expenditure masks low physical activity in obesity.* Int J Obes (Lond), 2013. **37**(7): p. 1006-11.

42. Prentice, A.M., A.E. Black, W.A. Coward, H.L. Davies, G.R. Goldberg, P.R. Murgatroyd, et al., *High levels of energy expenditure in obese women.* British Medical Journal (Clinical research ed.), 1986. **292**(6526): p. 983-987.

43. Bankoski, A., T.B. Harris, J.J. McClain, R.J. Brychta, P. Caserotti, K.Y. Chen, et al., *Sedentary activity associated with metabolic syndrome independent of physical activity.* Diabetes Care, 2011. **34**(2): p. 497-503.

44. Yadav, R.L., P.K. Yadav, L.K. Yadav, K. Agrawal, S.K. Sah, and M.N. Islam, *Association between obesity and heart rate variability indices: an intuition toward cardiac autonomic alteration - a risk of CVD.* Diabetes Metab Syndr Obes, 2017. **10**: p. 57-64.

45. Rossi, R.C., L.C.M. Vanderlei, A.C.C.R. Gonçalves, F.M. Vanderlei, A.F.B. Bernardo, K.M.H. Yamada, et al., *Impact of obesity on autonomic modulation, heart rate and blood pressure in obese young people.* Autonomic Neuroscience, 2015. **193**: p. 138-141.

46. Itagi, A.B., N. Ss, S. Bondade, and Y. G Y, *Relationship of resting heart rate with body composition and obesity among young adults in India - A cross sectional study.* National Journal of Physiology, Pharmacy and Pharmacology, 2014. **4**: p. 1.

47. Aneni, E.C., E.T. Oni, C.U. Osondu, S.S. Martin, M.J. Blaha, E. Veledar, et al., *Obesity modifies the effect of fitness on heart rate indices during exercise stress testing in asymptomatic individuals.* cardiology, 2015. **132**(4): p. 242-248.

48. Zhang, S.Y., J.H. Wu, J.W. Zhou, Z. Liang, Q.Y. Qiu, T. Xu, et al., *Overweight, resting heart rate and prediabetes/diabetes: A population-based prospective cohort study among Inner Mongolians in China.* Scientific Reports, 2016. **6**(1): p. 23939.

49. Freitas Júnior, I.F., P.A. Monteiro, L.S. Silveira, S.U. Cayres, B.M. Antunes, K.N. Bastos, et al., *Resting heart rate as a predictor of metabolic dysfunctions in obese children and adolescents.* BMC Pediatrics, 2012. **12**(1): p. 5.

50. Shigetoh, Y., H. Adachi, S.-i. Yamagishi, M. Enomoto, A. Fukami, M. Otsuka, et al., *Higher Heart Rate May Predispose to Obesity and Diabetes Mellitus: 20-Year Prospective Study in a General Population.* American Journal of Hypertension, 2009. **22**(2): p. 151-155.

51. Reimers, A.K., G. Knapp, and C.D. Reimers, *Effects of Exercise on the Resting Heart Rate: A Systematic Review and Meta-Analysis of Interventional Studies.* J Clin Med, 2018. **7**(12).

52. Carter, J.B., E.W. Banister, and A.P. Blaber, *Effect of Endurance Exercise on Autonomic Control of Heart Rate.* Sports Medicine, 2003. **33**(1): p. 33-46.

53. Arts, F.J. and H. Kuipers, *The relation between power output, oxygen uptake and heart rate in male athletes.* Int J Sports Med, 1994. **15**(5): p. 228-31.

54. Hills, A.P., N.M. Byrne, and A.J. Ramage, *Submaximal markers of exercise intensity.* J Sports Sci, 1998. **16 Suppl**: p. S71-6.

55. Li, S.S.W., O.H.T. Chan, T.Y. Ng, L.H. Kam, C.Y. Ng, W.C. Chung, et al., *Gender Differences in Energy Expenditure During Walking With Backpack and Double-Pack Loads.* Human Factors, 2018. **61**(2): p. 203-213.

56. Kravitz, L., R.A. Robergs, V.H. Heyward, D.R. Wagner, and K. Powers, *Exercise mode and gender comparisons of energy expenditure at self-selected intensities.* Medicine and science in sports and exercise, 1997. **29**(8): p. 1028-1035.

57. Keim, N.L., A.Z. Belko, and T.F. Barbieri, *Body Fat Percentage and Gender: Associations with Exercise Energy Expenditure, Substrate Utilization, and Mechanical Work Efficiency.* International Journal of Sport Nutrition, 1996. **6**(4): p. 356-369.

58. *2018 Standard Occupational Classification System* 2018; Available from: https://www.bls.gov/soc/2018/major_groups.htm.

LIST OF TABLES

Table 1A –Age and anthropometric data of participants. All the data are reported as mean ± standard deviation.

		Age [y.o.]	BMI [kg/m^2]
**Normal BMI (n=10)**	**Male (n=5)**	28.2 ± 8.9	23.0 ± 1.1
	**Female (n=5)**	31.2 ± 12.7	22.0 ± 2.7
	**Subtotal**	29.7 ± 11.1	22.5 ± 2.1
**Overweight BMI (n=10)**	**Male (n=5)**	32.8 ± 12.8	26.6 ± 1.5
	**Female (n=5)**	42.2 ± 11.4	27.2 ± 0.7
	**Subtotal**	37.5 ± 13.0	26.9 ± 1.2
**Obese BMI (n=10)**	**Male (n=5)**	26.2 ± 4.9	40.5 ± 9.3
	**Female (n=5)**	34.6 ± 13.8	31.5 ± 1.3
	**Subtotal**	30.4 ± 11.2	36.1 ± 8.0

Table 1B – Summary of subjects' race and ethnicity. All the data are reported as number of subjects and percentages based on the total (n=30). Data are compared to Miami-Dade County demographics as per 2020 Census.

	Hispanic or Latino	White alone, not Hispanic or Latino	Black or African American	Asian	Two or more races
**Male (n=15)**	6 (20.0%)	3 (10.0%)	1 (3.3%)	4 (13.3%)	1 (3.3%)
**Female (n=15)**	6 (20.0%)	2 (6.7%)	2 (6.7%)	1 (3.3%)	4 (13.3%)
**Subtotal**	12 (40.0%)	5 (16.6%)	3 (10%)	5 (16.6%)	5 (16.6%)
**Miami-Dade County 2020 Census** [21]	***69.1%***	***13.6%***	***17.4%***	***1.6%***	***1.3%***

Table 1C – Summary of subjects' occupations. All the data are reported as number of subjects, and standard occupational classifications are based on the U.S. Bureau of Labor Statistics [58].

Standard occupational classification	Title	Number of subjects	Total
Educational instruction and library	Graduate student	5	16
	Research associate	2	
	Teacher	1	
	Undergraduate student	8	
Healthcare practitioners and technical	Nurse	1	2
	Physical therapist	1	
Management, Sales and related	Cashier	4	6
	Nonprofit manager	1	
	Sales	1	
Service Industry	Appraiser	1	6
	Driver	1	
	Engineer	1	
	Janitor	1	
	Line cook	1	
	Translator	1	

Table 2 – Summary of RNLE components. The RNLE computes the RWL for a manual lifting task based on a specific variable that reflects the work geometry and lifting frequency of that particular task. These input values correspond to a RLNE multiplier, which is directly used in the RWL calculation [18].

RNLE: $RWL = LC \times HM \times VM \times DM \times AM \times FM \times CM$			
**Variable**	**Input Value**	**RNLE Component**	**RWL**
Load Constant (LC)		51 lbs.	
Horizontal Multiplier (HM)	10”	1	
Vertical Multiplier (VM)	5”	0.81	27.5 lbs
Distance Multiplier (DM)	25”	0.89	
Asymmetry Multiplier (AM)	No trunk rotation	1	
Frequency Multiplier (FM)	6 lifts/min over 20 min	0.75	
Coupling Multiplier (CM)	Good	1	

Table 3 – Metabolic variables versus BMI and gender. Data are reported in terms the average value at the steady state of VCO_2, VO_2, RER, HR, and EER in comparison to a subject's BMI classification and gender. In particular, marginal means for BMI classifications are grouped using a Fisher Pairwise comparison. A p-value for the ANOVA test comparing each metabolic variable with BMI class is also reported, with (*) indicating statistically significant difference.

		**Normal (n=10)**	**Overweight (n=10)**	**Obese (n=10)**	**Subtotal (n=30)**	**P-Value**
**VCO_2 (mL/min)**	**Male (n=15)**	680.9	684.5	1060.3	***808.6***	0.000*
	**Female (n=15)**	593.3	744.1	814.6	***717.3***	
	**Subtotal (n=30)**	***637.1***	***714.3***	***937.4***	***762.9***	
	**Grouping**	**B**	**B**	**A**		
**VO_2 (mL/min)**	**Male**	821.7	886.9	1279.5	***996.0***	0.000*
	**Female**	695.7	869.6	998.1	***854.5***	
	**Subtotal**	***758.7***	***878.2***	***1138.8***	***925.3***	
	**Grouping**	**B**	**B**	**A**		
**RER**	**Male**	0.83	0.78	0.83	***0.81***	0.679
	**Female**	0.86	0.86	0.82	***0.84***	
	**Subtotal**	***0.84***	***0.82***	***0.82***	***0.83***	
	**Grouping**	**A**	**A**	**A**		
**HR (BPM)**	**Male**	109.6	110.7	143.3	***121.2***	0.012*
	**Female**	124.5	123.2	137.7	***128.5***	
	**Subtotal**	***117.0***	***117.0***	***140.5***	***124.8***	
	**Grouping**	**B**	**B**	**A**		
**EER (kcal/min)**	**Male**	4.1	4.3	6.3	***4.9***	0.000*
	**Female**	3.4	4.2	4.9	***4.2***	
	**Subtotal**	***3.8***	***4.3***	***5.6***	***4.5***	
	**Grouping**	**B**	**B**	**A**		

LIST OF FIGURES

Figure 1. Geometrical setting for experiment. Subjects stood in front of a 27.5 pound box (10”x10”x10”) and moved it from the floor to the 30” high table, and from the table to the floor at a frequency of six lifts per minute for 20 minutes. The hand grasps of the box were 10” away from the midpoint of the subject’s ankles and 5” from the floor. A ‘freestyle’ lifting technique was allowed. The subject wore a mask that connected to the CardioCoachCO_2 metabolic cart to measure VCO_2 and VO_2 and a heart rate monitor for the duration of the lifting activity.

Figure 2. Time-dependent metabolic parameters across testing period. Subjects were grouped into their corresponding BMI classifications and gender: black circle is obese male, black triangle is obese female, grey circle is overweight male, grey triangle is overweight female, white circle is normal male, and white triangle is normal female. An average value for five metabolic dependent variables was taken for each BMI-gender group over the testing period. The figure represents the relationship between elapsed time and average: (A) VCO_2; (B) VO_2; (C) RER; (C) HR; and (D) EER, with the dotted line representing the safety limit (4.7 kcal/min).

Figure 3. Relationships between metabolic variables and BMI. Each circle represents a subject, and the colors of the circles correspond to the BMI of that subject: black is obese, grey is overweight, and white is normal. An average steady state value for each metabolic variable was graphed versus each subject’s BMI. Dashed lines reflect the linear regressions, and R^2 and p-values are provided for the data of each metabolic variable. The figure represents the relationship between a subject’s BMI and average steady state: (A) VCO_2; (B) VO_2; (C) RER; (D) HR; and (E) EER, with the dotted line representing the safety limit (4.7 kcal/min).

Figure1

Click here to access/download;Figure;figure 1.jpg

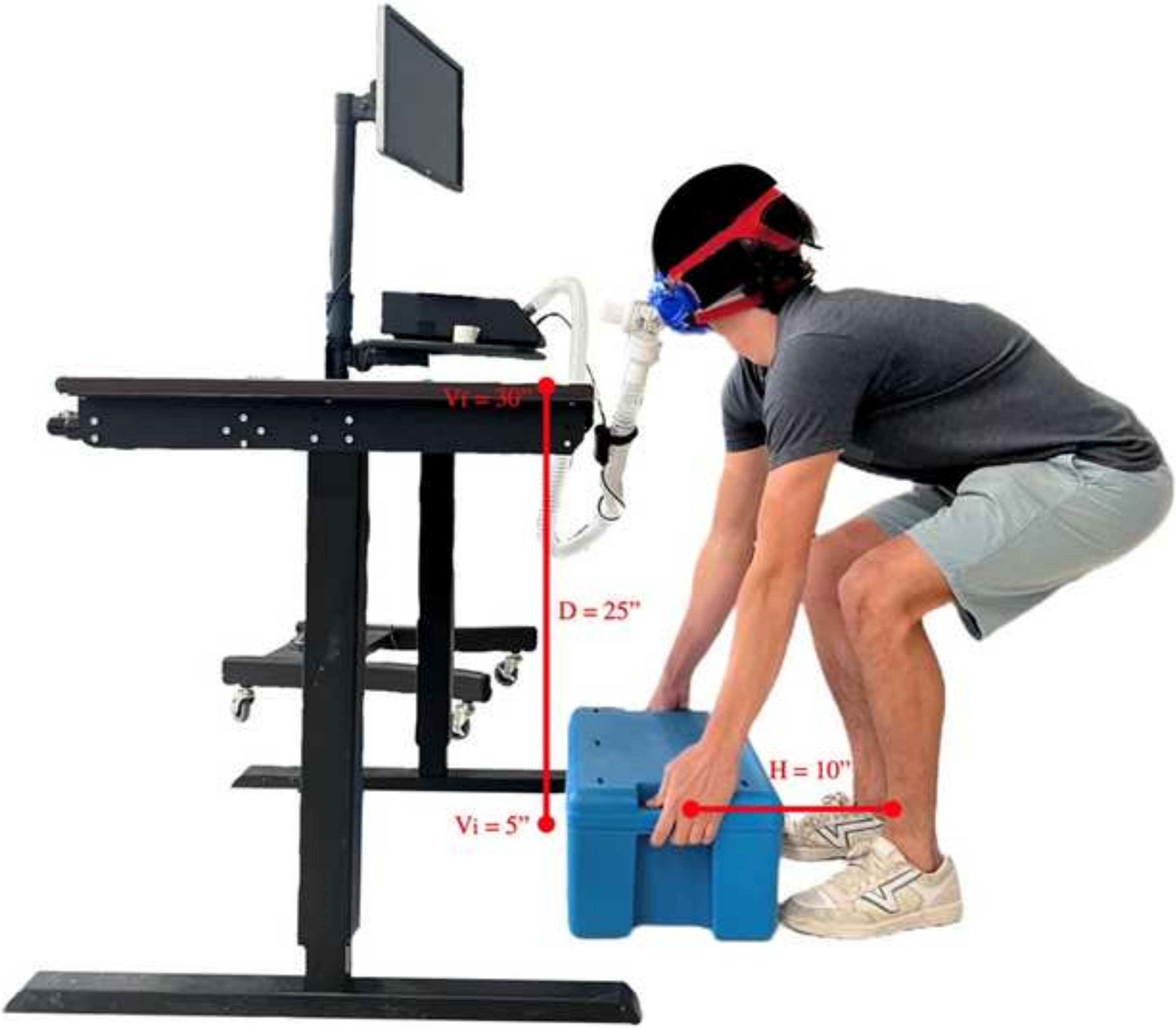

Figure2a

Click here to access/download;Figure;Figure 2a.jpg

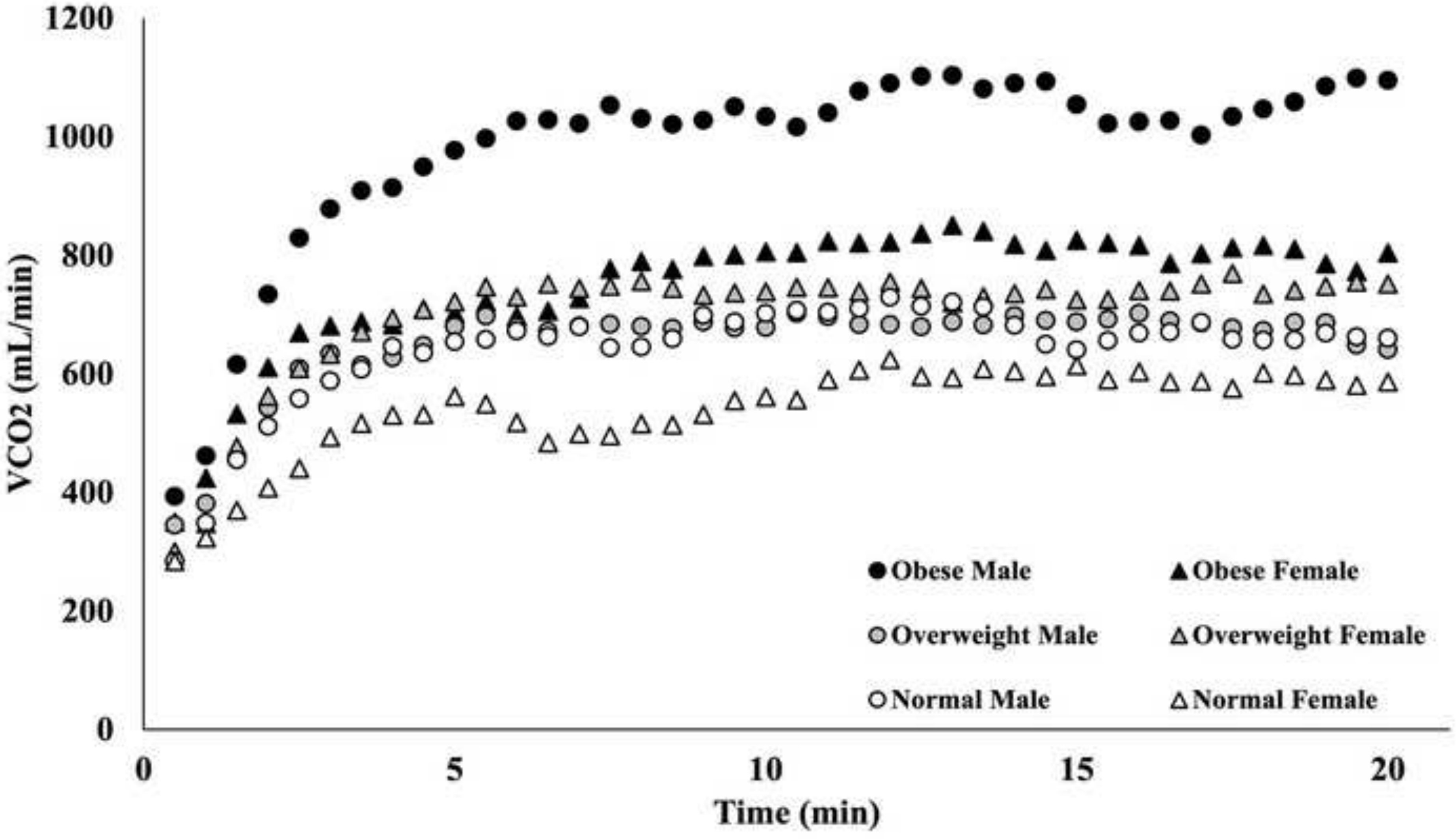

Figure2b

Click here to access/download;Figure;Figure 2b.jpg

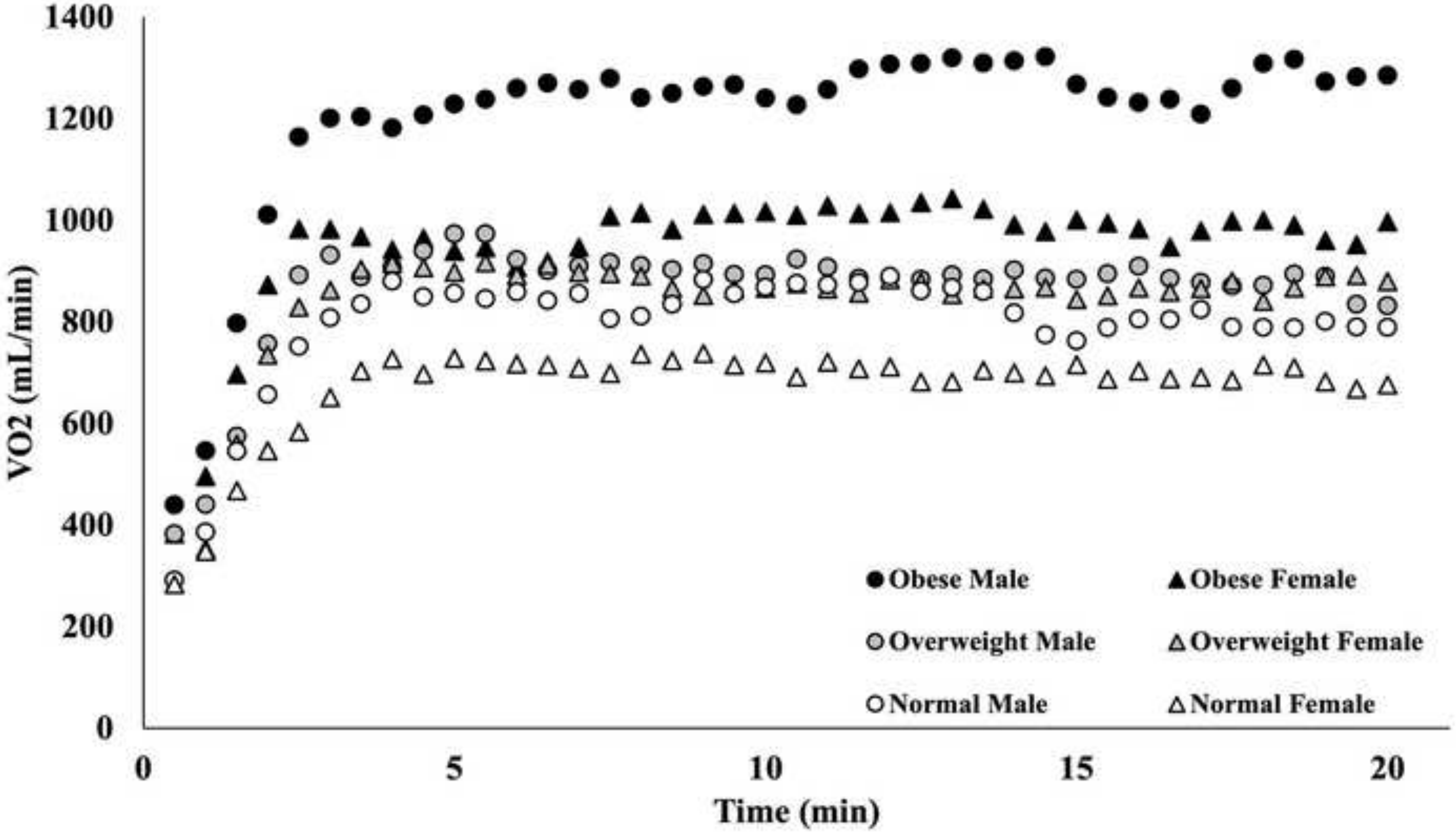

Figure2c

Click here to access/download;Figure;Figure 2c.jpg

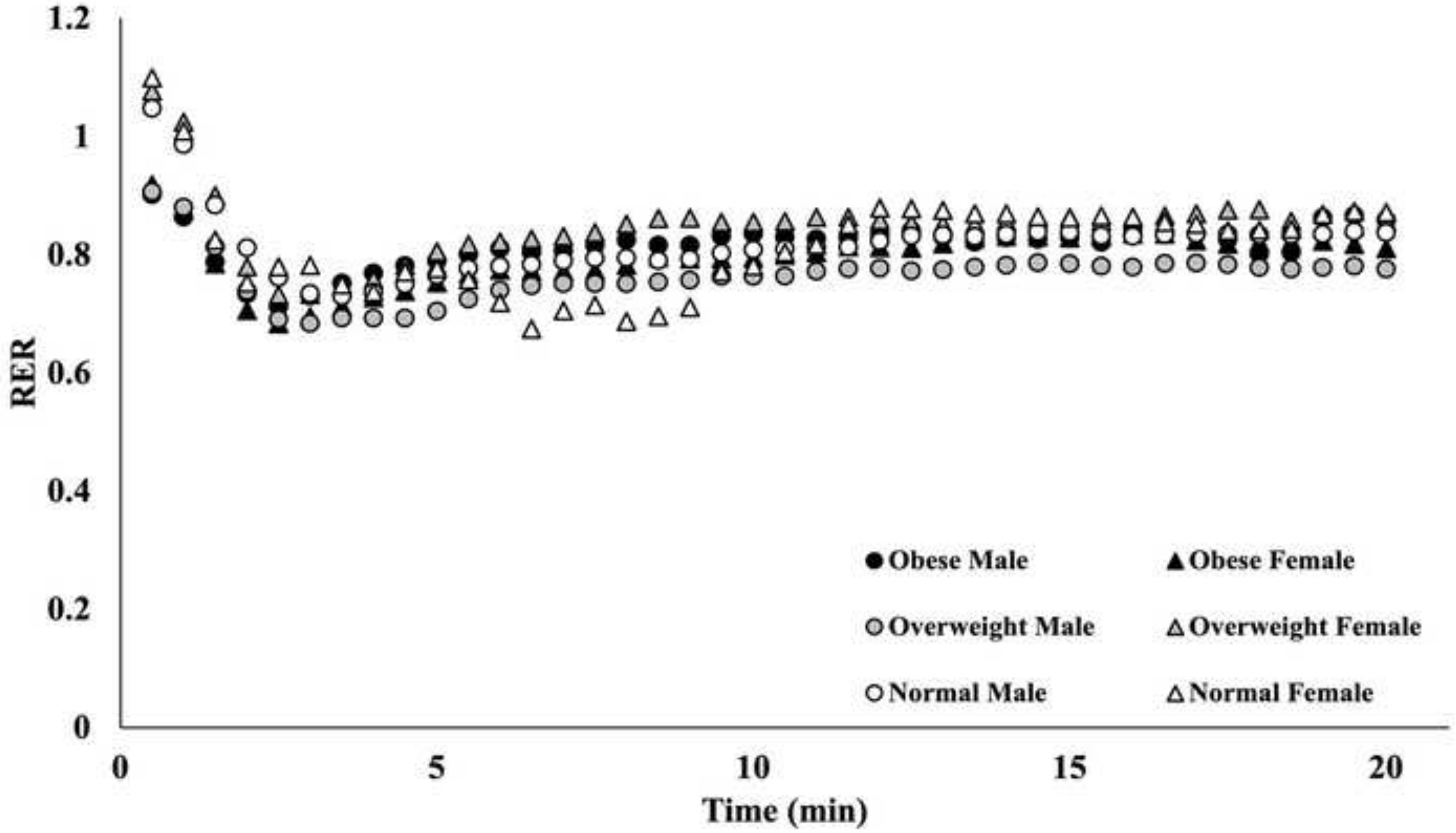

Click here to access/download;Figure;Picture2d.jpg

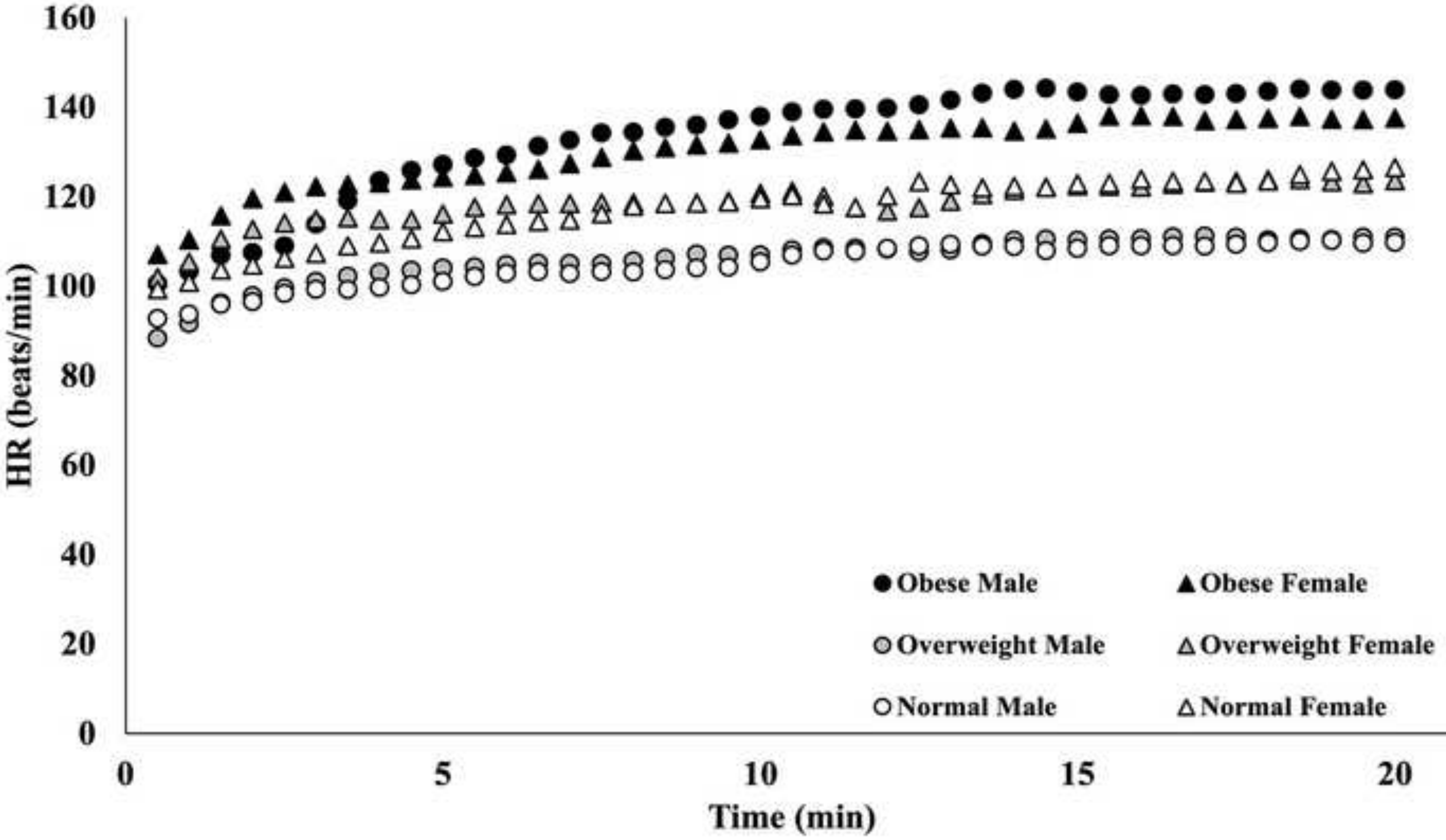

Figure2e

Click here to access/download;Figure;Picture2e.jpg

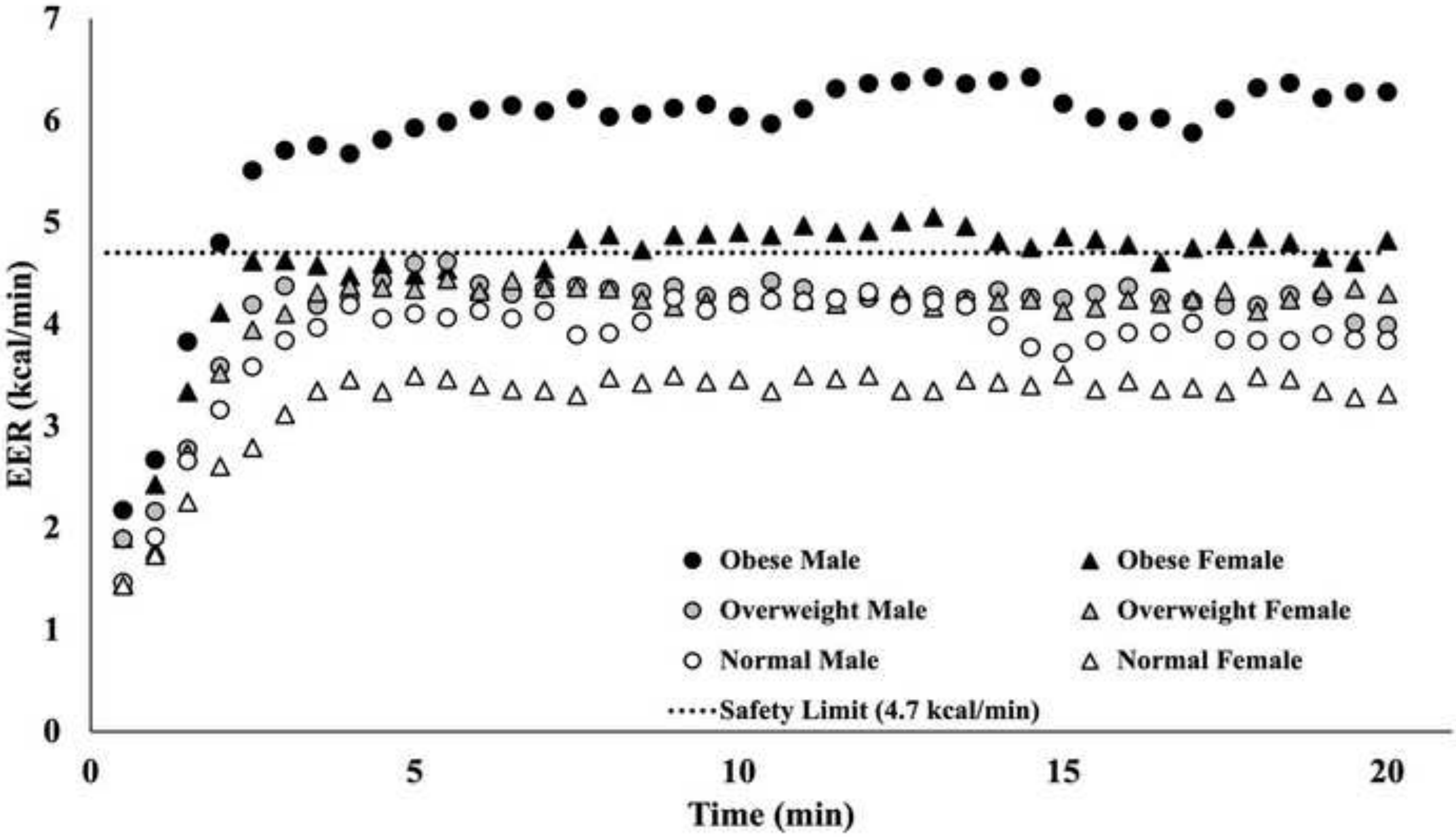

 Click here to access/download;Figure;Figure 3a.jpg

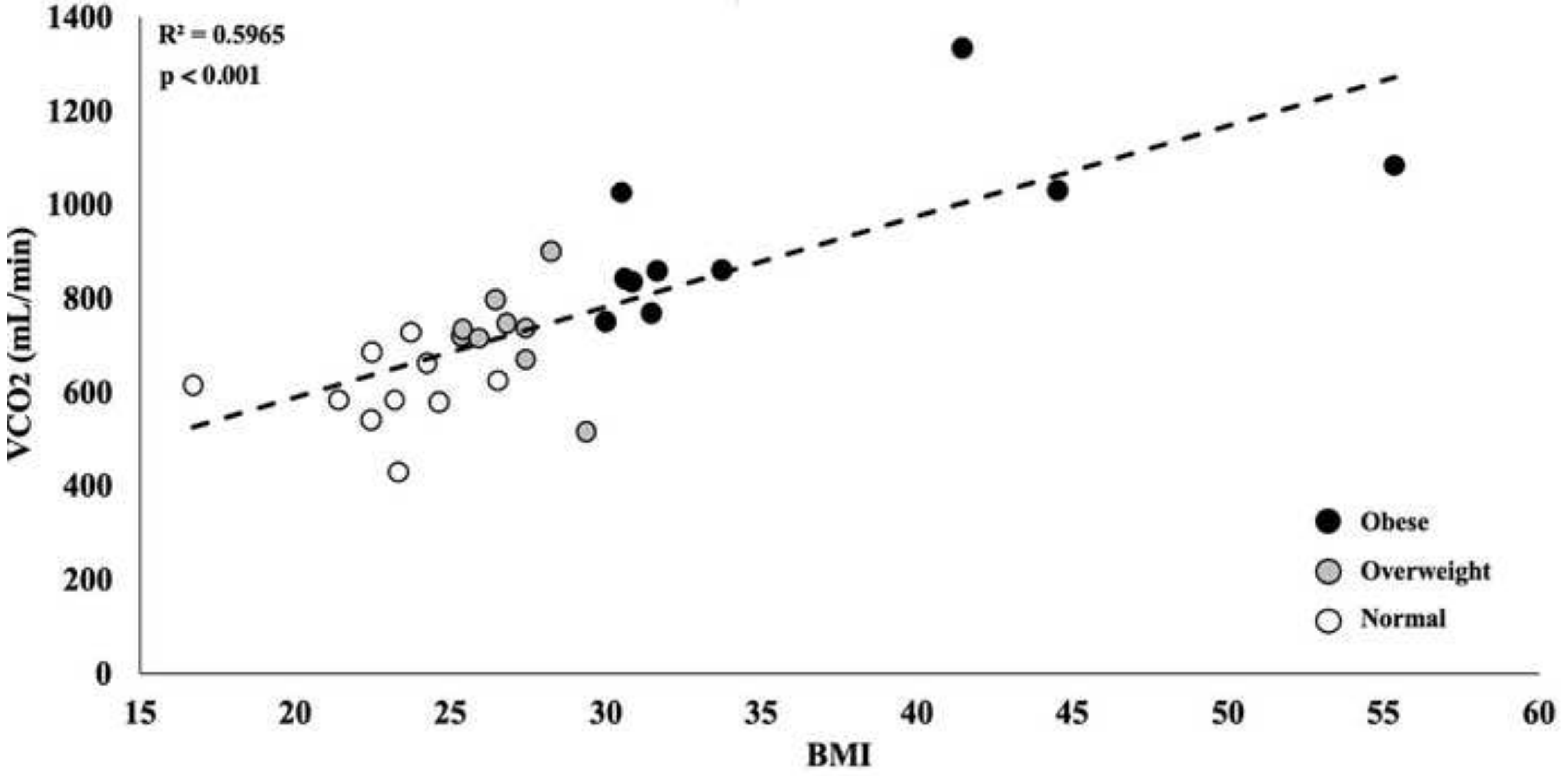

 Click here to access/download;Figure;Figure 3b.jpg

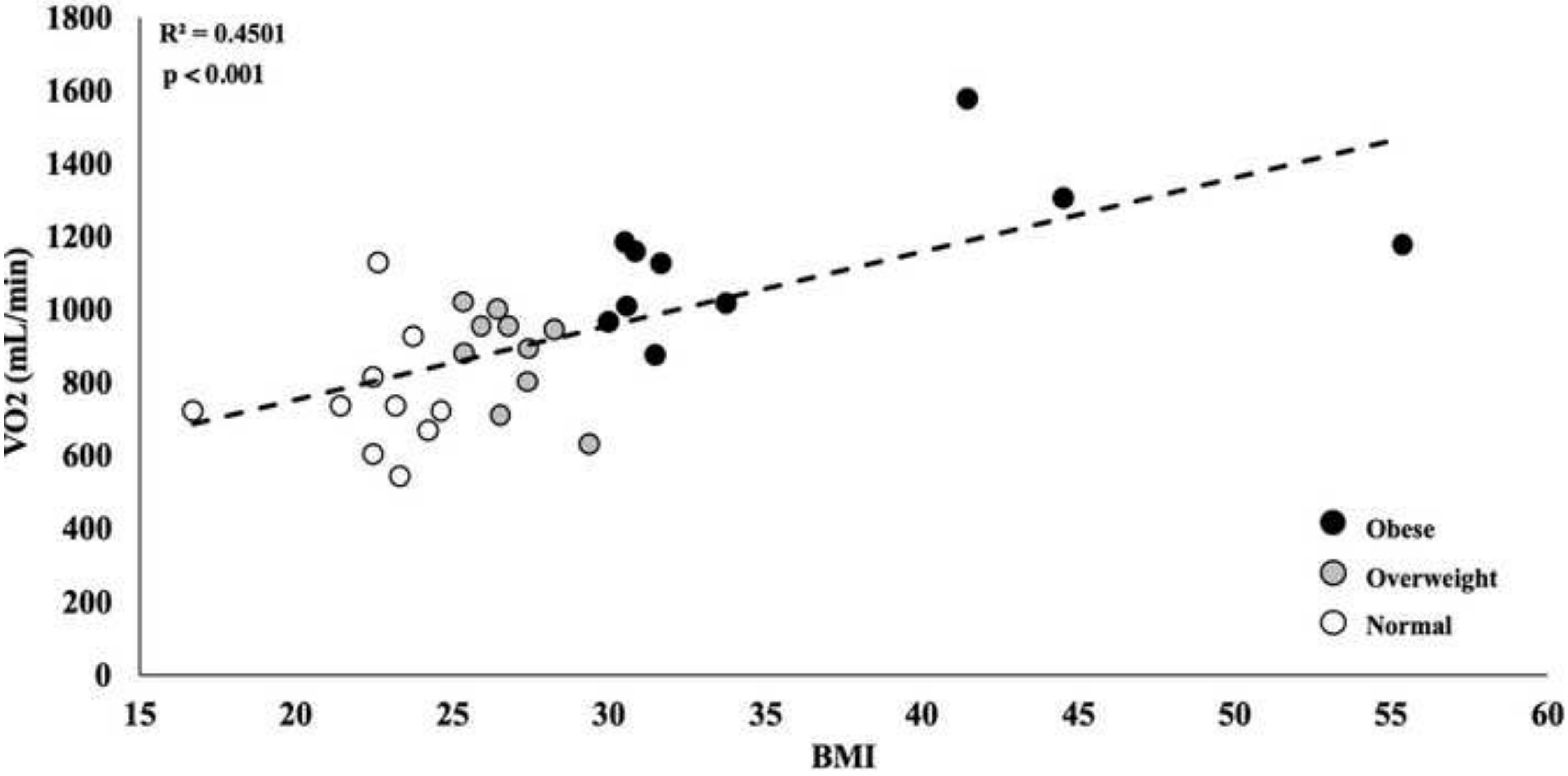

 Click here to access/download;Figure;Figure 3c.jpg

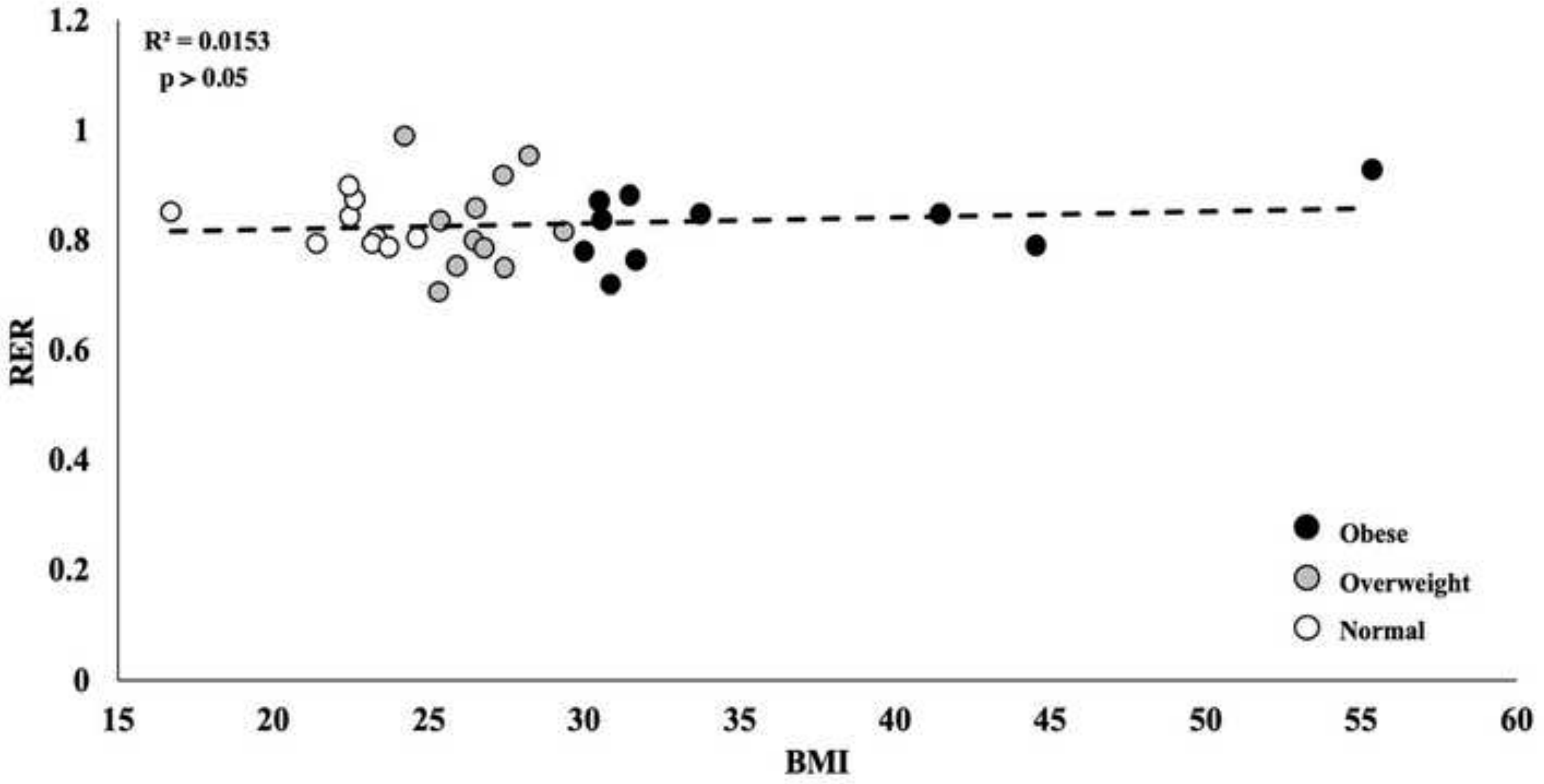

Figure
Click here to access/download;Figure;Figure 3d.jpg

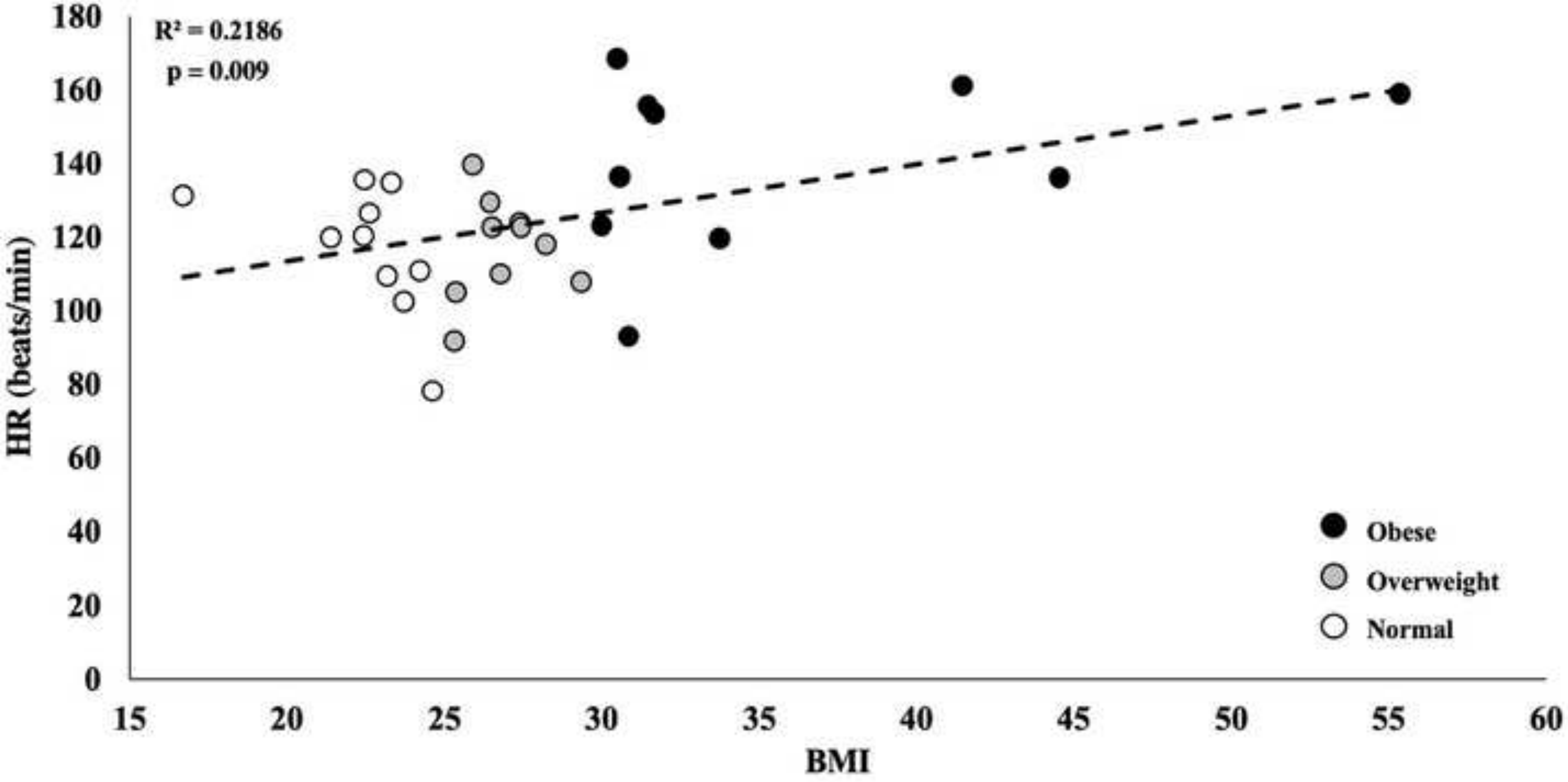

Figure Click here to access/download;Figure;Figure 3e.jpg

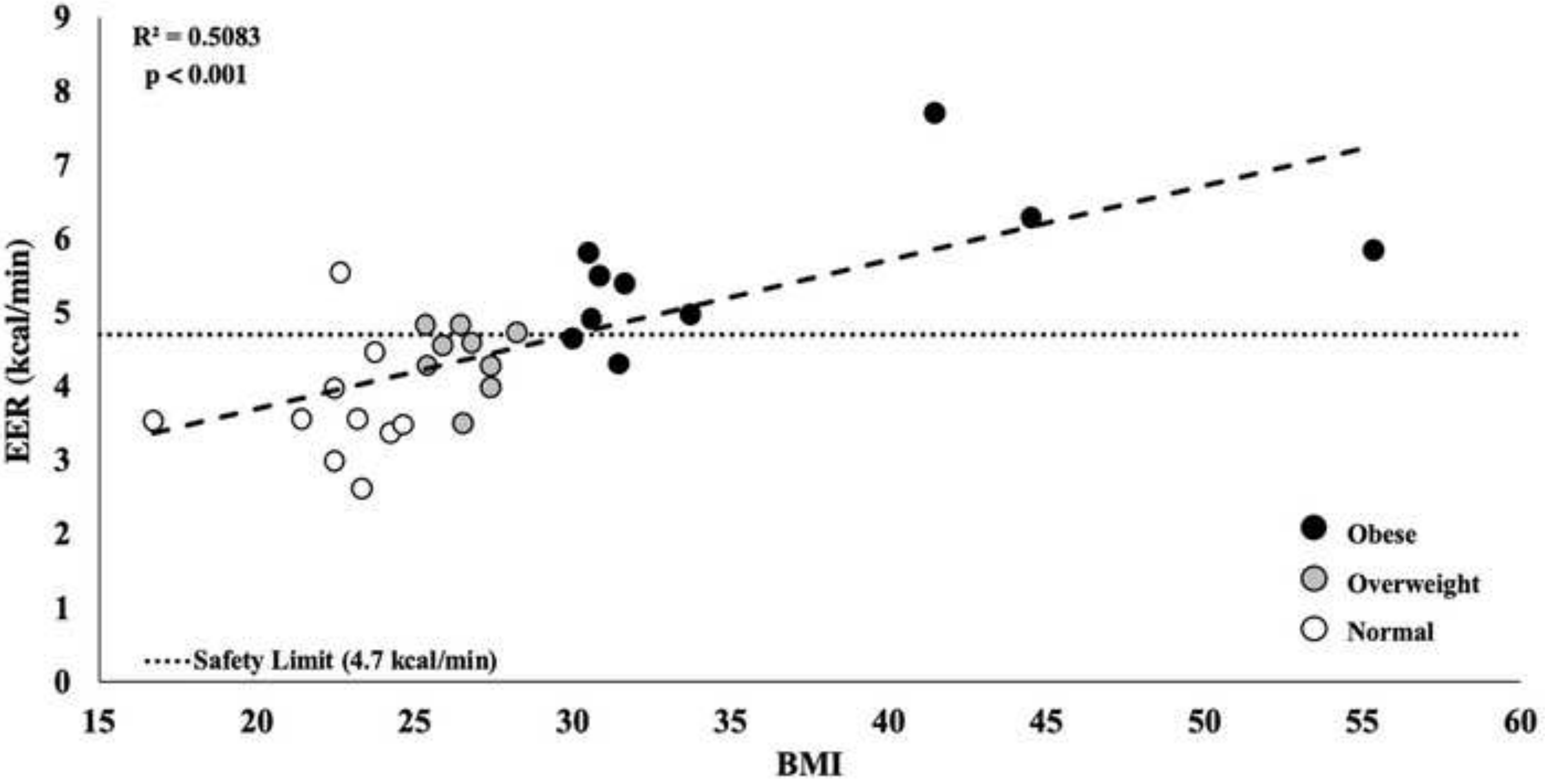

Supplement: Attachment — Submitted filename: PONE-D-22-28339 review.pdf [file pone.0278858.s002.pdf]
